# Novel Nanostructured Pd/Co-Alumina Materials for the Catalytic Oxidation of Atmospheric Pollutants

**DOI:** 10.3390/nano14010124

**Published:** 2024-01-04

**Authors:** Eleni F. Iliopoulou, Eleni Pachatouridou, Angelos A. Lappas

**Affiliations:** Chemical Process and Energy Resources Institute, Centre for Research and Technology Hellas (CPERI), CERTH, Thermi, GR-57001 Thessaloniki, Greece; e_pahat@certh.gr (E.P.); angel@certh.gr (A.A.L.)

**Keywords:** CO oxidation, methanol oxidation, spray impregnation, core–shell, cobalt oxide, palladium

## Abstract

Cobalt-doped alumina catalysts were prepared using different methods, either conventional wet impregnation (WI) and/or advanced spray impregnation (SI), and they were evaluated as novel oxidation catalysts for CO and MeOH oxidation. The spray impregnation technique was used with the aim of achieving the synthesis of core–shell catalytic nanostructures to secure the chemical/thermal stability of active sites on the catalyst carrier. The catalysts were further promoted with a low Pd content (0.5 wt.%) incorporated via either incipient wetness impregnation (DI) or spray impregnation. The results revealed the superior performance of the spray-impregnated catalysts (Co/γ-Al_2_O_3_-SI) for both reactions. The deposition of Co oxide on the outer surface of the alumina particle (SEM images) and the availability of the active Co phase resulted in the enhancement of the Co/γ-Al_2_O_3_ catalysts’ oxidation activity. Pd incorporation increased the catalysts’ reducibility (TPR-H_2_) and improved the catalysts’ performance for both reactions. However, the Pd incorporation method affected the catalytic performance; with the SI method, the active phase of Co_3_O_4_ was probably covered with PdO and was not available for the oxidation reactions. On the contrary, the incorporation of Pd with the DI method resulted in a better dispersion of PdO all over the Co/Al catalyst surface, maintaining available Co active sites and a better Pd-Co interaction. MeOH desorption studies revealed the methanol oxidation mechanism: the Co/Al catalysts promoted the partial oxidation of MeOH to formaldehyde (HCHO) and dehydration to dimethyl ether (DME), while the Pd-based Co/Al catalysts enhanced the complete oxidation of methanol to CO_2_ and H_2_O.

## 1. Introduction

The catalytic abatement of CO and volatile organic compound (VOC) emissions from either transport or the industrial sector have been the target of many ongoing research studies due to strong environmental concerns and increasingly stricter legislation. However, methanol is recently gaining more and more interest as an environmentally friendly fuel for either gasoline or diesel engines, as it is cheap, has a high-octane value, has a large latent heat of vaporization, is sustainable and has good combustion properties (a high oxygen content and a low ratio of carbon/hydrogen, as well as being non-shooting), and it is thus considered a promising alternative [1,2]. Moreover, methanol combustion/utilization in vehicles may decrease the emission of undesired pollutants, including CO, unburned hydrocarbons, particulate matter and nitrogen oxides, emitting, however, other harmful partial oxidation products, such as formaldehyde and unburned methanol vapor [3]. 

Supported noble metal catalysts are well known for their high activity in the catalytic CO oxidation or deep methanol oxidation reactions, while in the latter case, they also exhibit higher selectivity, presenting the lowest formaldehyde (HCHO) yield [4,5]. However, their high cost inhibits their final development and application. Besides noble metals, transition metal oxides have also been explored and reviewed as catalysts for VOC oxidation most commonly supported on γ-Al_2_O_3_ [6,7]. Most research groups have mainly focused on active metal oxide components; however, the type of catalytic carrier is also important, as it crucially affects the generation of active sites and, thus, the catalytic efficiency [8]. Luo et al. [3] investigated the promoter effect of Ce_x_Zr_1−x_O_2_ on Al_2_O_3_ supports, which led to the development of the best Pd-based catalyst presenting the optimum methanol oxidation with minimum HCHO formation at a low temperature. The promoting effect was attributed to the enhanced interaction between Pd and the modified support resulting in more reducible species. Moreover, more activated surface oxygen and stronger basic properties are also beneficial for methanol oxidation.

The screening of a series of Μ/γ-Al_2_O_3_ catalytic systems (1.0 wt.% of metals M: Cu, Mn, Ce, K, Ag, Cu–Mn, Cu–Ce, Cu–Ag and Cu–K) was also performed for methanol oxidation, revealing the superiority of silver-based catalysts, which was attributed to the dispersion of Ag species on alumina supports [9]. Cu-containing catalysts have also been explored for VOC oxidation, including methanol, presenting enhanced catalytic activity when adding 0.5 and 1.0 wt.% of a potassium promoter. Other reported promoters for Cu catalysts include manganese [10], ceria [11] and Ti. With regard to Ti, doping a Pd-Cu/CeO_2_ catalyst with titanium promotes the metal–support interaction, subsequently leading to more oxidized palladium species and surface oxygen vacancies on the catalyst and thus achieving improved catalytic activity (T_50_ and T_90_ of methanol oxidation are 88 °C and 138 °C, respectively) [12]. In all cases, varying synthesis approaches, different catalytic carriers and operating conditions are additional parameters crucially affecting the final characteristics and, thus, the final catalytic efficiency.

However, among the transition metals, Co_3_O_4_ materials are reported as the most effective catalysts for the oxidation of CO, due to both their weak oxygen bond strength and their high redox capacity, and for this reason, they have been extensively studied [13,14]. In addition, there has been an impressive recent increase in studies on morphology-dependent nano-catalysis, as the synthesis method seems to have a crucial effect on the derived structural properties, such as the specific surface area, the surface oxidation state, the strength of the interaction with the reactants and, finally, the dispersion of the active species. Cobalt catalysts usually involve nano-sized Co oxide particles (+2 and +3) with various shapes and crystal structures, while the Co^+3^ ratio is very important and directly related to the formation of oxygen vacancies. The catalytic efficiency of various cobalt nanoparticles in the CO oxidation reaction seems to follow the order of nanoplates > nanorods > nanocubes > nanosphere, while various mechanisms have been proposed for the catalytic oxidation of CO. A recent study provided a good review of pure and supported cobalt catalysts, their preparation methods and their application in CO emission control [15]. In order to enhance their catalytic performance, the active phase can also be dispersed, in various loadings, on a substrate with a large specific surface, such as γ-Al_2_O_3_. Such nanorod-structured Co_3_O_4_/Al_2_O_3_ catalysts were subjected to reductive–oxidative pretreatment, which led to surface reconstruction, reduced the size of Co_3_O_4_ crystallites and increased the Co percentage, bringing about a significant improvement in the catalytic activity [16]. Recently, Co-alumina oxidation catalysts were also prepared via the dynamic hydrothermal method and were further promoted with noble metals (1% Ru or Pd). The incorporation of Co into alumina imparts redox properties to the thermally stable but non-reducible alumina, creates strong substrate metal interaction effects (strong metal–support interactions) and, thus, facilitates the activation of oxygen from the gas phase [17]. In another recent study, Co_3_O_4_/a-Al_2_O_3_ catalysts were also prepared by applying the rotary chemical vapor deposition (CVD) method, aiming to develop nanoparticles with a very large dispersion on the surface of the catalytic substrate, thus achieving high activity in the catalytic oxidation of CO with the minimum percentage of Co_3_O_4_ content [14]. It is generally accepted that nanoparticles with a high specific surface area/volume ratio enhance catalytic performance by offering more available active sites, while this applies to both Co_3_O_4_ and noble metal (e.g., Pd) particles. To systematically interpret this effect of metal nanoparticles on catalytic activity, a series of catalysts were prepared in a recent study by controlling the size of Pd nanoparticles on a Co_3_O_4_ substrate by varying the calcination temperature. This approach fine-tuned the Pd particle size from 2.5 to 10.6 nm, demonstrating that size is a dominant factor in CO oxidation catalytic performance, which improves as Pd particles become smaller [18].

The present study concerns the preparation of innovative Co/alumina catalytic mat-rials (promoted or not with a low percentage of Pd), with the aim of enhancing catalytic activity in the oxidation of CO and methanol by improving their redox behavior and their high ability to store oxygen, mainly by varying the dispersion of both Co_3_O_4_ and Pd particles. Combining Pd with Co aims to reduce the amount of Pd and simultaneously enhance catalytic performance, as similarly reported [19,20,21,22,23] when, e.g., combining Pd and Cu. The evaluation of the catalytic materials concerns the catalytic oxidation of both CO (in a temperature range of 150–500 °C) and methanol (in a temperature range of 30–300 °C). 

## 2. Materials and Methods

All Co/γ-Al_2_O_3_ catalysts were prepared by applying either the conventional wet impregnation method (WI) or the advanced spray impregnation (SI) technique. Cobalt acetate tetrahydrate (Co(CH_3_CO_2_)_2_.4H_2_O, purchased from Sigma- Aldrich Chemie GmbH, Taufkirchen, Germany, purity > 99%) was used as the Co precursor salt, and two different cobalt loadings (1 wt.%, 5 wt.%) were incorporated on γ-Al_2_O_3_ (purchased from Saint-Gobain NorPro, Stow, OH, USA). When applying the conventional wet impregnation method, aqueous solutions of the precursor salt and alumina carrier were mixed and stirred at 72 °C for 1 h in a rotary evaporator (without vacuum). After that, the temperature was increased to 82 °C, and the water was evaporated under vacuum. The solid material was dried at 110 °C overnight and then calcined at 500 °C for 5 h under air flow. 

When applying the spray impregnation technique, the alumina carrier was initially loaded on a SI apparatus (Romace Innojet Ventilus V2/2.5, Steinen, Germany) and preheated at 100 °C, and then an aqueous solution of the Co precursor was sprayed on the alumina carrier. The derived solid material was dried at 110 °C overnight and then calcined at 500 °C for 5 h under air flow. The samples prepared with the wet impregnation method are labeled herein as xCo/Al-WI, while those prepared with the spray impregnation are labeled as xCo/Al-SI, where *x* is the Co loading (1 or 5 wt.%).

The addition of 0.5 wt.% Pd to Co/γ-Al_2_O_3_ samples was also performed, always after Co incorporation, by applying either the incipient wetness impregnation method (DI) and/or the spray impregnation method (SI) and always using Pd nitrate (Pd(NO_3_)_2_·2H_2_O, Sigma-Aldrich Chemie GmbH, Taufkirchen, Germany) as the precursor salt. The derived samples were dried at 110 °C overnight and calcined under air flow at 500 °C for 5 h. The code names of the Pd-incorporated samples are Pd(y)-xCo/Al-z, where *y* is either DI or SI (method of Pd incorporation), *x* is the Co loading (1 wt.%, 5 wt.%), and *z* is either WI or SI (method of Co incorporation into γ-Al_2_O_3_ support). The particle size of all catalytic material was 60–90 μm.

Textural and structural characterization was performed for all catalytic materials: Inductively Coupled Plasma–Atomic Emission Spectroscopy (ICP-AES) was used for the determination of the Co and Pd content (wt.%) in a 4300 DV PerkinElmer Optima spectrometer. Powder X-ray diffraction (XRD) measurements were performed for the determination of the metal oxide phases formed (Co, Pd), using a SIEMENS D-500 diffractometer, employing CuKa1 radiation (λ = 0.15405 nm) and operating at 40 kV and 30 mA. The XRD patterns were accumulated in the range of 10–80° 2θ, every 0.02° (2θ), with a counting time of 2 s per step. N_2_ adsorption/desorption experiments were performed for the determination of porous characteristics and mainly the surface area (BET method). The experiments were conducted at −196 °C, using an Automatic Volumetric Sorption Analyzer (Nova 2200e Quantachrome flow apparatus, Boynton Beach, FL, USA). Beforehand, the samples were outgassed overnight at 250 °C under vacuum. SEM imaging was performed using a JEOL JSM-IT500 microscope to explore the morphological characteristics. EDS spectra and mapping were obtained using an Oxford Instruments x-Act detector. The samples were embedded in resin, which was grinded and polished, while each resin was gold-plated. The analysis was carried out by applying a voltage of 20 kV.

Temperature-programmed reduction with H_2_ (TPR-H_2_) was applied to determine the reducibility of the catalytic materials in a bench-scale TPX unit. TPR-H_2_ was performed by initially loading 150 mg of each catalytic sample in a quartz fixed-bed reactor, coupled with mass spectrometry (MS). Prior to the measurement, a pre-treatment under pure He flow was carried out from room temperature (RT) up to 400 °C (with a heating rate of 10 °C/min) for 1 h. Then, the sample was cooled down to RT and heated to 800 °C with a ramp of 10 °C/min under 5 vol.% H_2_/He. The reactor exit was coupled with MS, detecting the signal of H_2_ (*m*/*z* = 2). The temperature-programmed desorption of methanol (TPD-MeOH) was performed in the same unit. Initially, a pre-treatment under pure He flow was carried out (from RT up to 400 °C, for 1 h), and the sample was cooled down to RT. The sorption was performed at RT until the sample’s saturation with 0.1 vol.% CH_3_OH/He. The desorption was performed with (A) He and (B) 0.1 vol.% O_2_/He, starting from RT up to 500 °C (with a ramp of 10 °C/min). MS detected the signals of CH_3_OH (*m*/*z* = 31), dimethyl ether DME (*m*/*z* = 45), formaldehyde HCHO (*m*/*z* = 29) and CO_2_ (*m*/*z* = 44). The temperature-programmed desorption of CO_2_ (TPD-CO_2_) was performed to determine the basic properties of the studied catalysts in the same TPX unit. Prior to the measurement, a pre-treatment under He flow was carried out from RT up to 600 °C for 1 h. The sorption of CO_2_ was conducted by exposing the catalyst to 10 vol.% CO_2_/He at 70 °C for 1 h. The gaseous or physiosorbed CO_2_ was removed by purging with He flow at 70 °C for 1 h. Then, the sample was heated to 800 °C (ramp of 10 °C/min), under He. The desorbed CO_2_ (*m*/*z* = 44) was monitored continuously via mass spectrometry. 

The catalytic materials were evaluated for the CO and MeOH oxidation reaction in a quartz fixed-bed reactor. Concerning the CO oxidation experiments, the reactor was loaded with 0.6 g of catalyst, while the total gas flow feed rate was 900 cm^3^/min, corresponding to a gas hourly space velocity (GHSV) of 40,000 h^−1^. The feed composition was 1 vol.% CO and 1 or 10 vol.% O_2_, balanced with He, while the CO conversion was monitored in the 150–500 °C range (in a decreasing temperature mode). The composition of the effluent gas was analyzed using an FT-IR gas multi-analyzer from MKS instruments (MKS-MG2030 Germany). MeOH oxidation was performed in the same unit. In that case, the reactor was loaded with 0.4 g of catalyst, and the total gas flow feed rate was 600 cm^3^/min (preserving GHSV ~40,000 h^−1^). The feed composition was 0.1 vol.% CH_3_OH and 0.1 vol.% O_2_, balanced with He. Methanol conversion was monitored in the 30–300 °C range (in a decreasing temperature mode), and both methanol and the reaction products (H_2_O, CO_2_, dimethyl ether (DME) and formaldehyde (HCHO)) were analyzed using the same MKS FT-IR gas analyzer.

## 3. Results

### 3.1. Catalyst Characterization Results

#### 3.1.1. Textural Characterization (ICP, BET)

Table 1 presents the Co and Pd loadings and the textural properties (surface area, pore volume and size) of all catalysts under study. The ICP results revealed that the desired metal loadings (1 and 5 wt.% for Co/Al and 0.5 wt.% for Pd) were achieved in most cases; only for the 5Co/Al-WI sample (and consequently also for Pd(DI)-5Co/Al-WI) was a significant (9.2%) higher Co loading observed, while the Pd loadings were almost always (excluding the Pd(SI)-5Co/Al-SI sample) 16–20% higher than the nominal percentage. As expected, the incorporation of either Co or Pd metal slightly affected the textural properties of the alumina. The surface area of the oxide support gradually decreased due to the surface being partially covered by the low-surface-area clusters of the deposited active components. This explains the larger surface decrease for the 5 wt.% Co materials and the further slight decrease after successive Pd deposition. It is interesting to note, however, that no important differences in textural properties were observed when comparing the materials prepared using the different synthesis methods.

#### 3.1.2. Structural Characterization (XRD, SEM)

**Figure 1:**
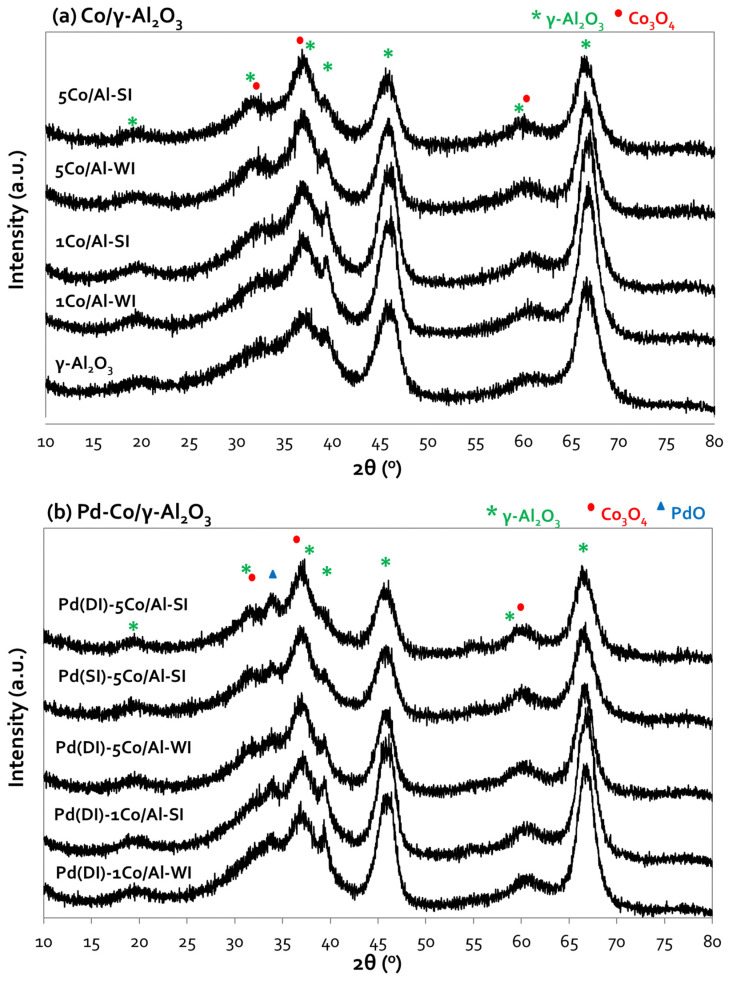
X-ray diffractograms of (**a**) Co/γ-Al_2_O_3_ and (**b**) Pd-based Co/γ-Al_2_O_3_ samples, prepared with different methods and various Co loadings.

The XRD patterns of the Co/Al and Pd-based Co/Al samples are shown in Figure 1a,b, respectively. The diffractogram of bare alumina (Figure 1a) exhibits the peaks of γ-Al_2_O_3_ at 2θ: 38°, 46° and 67° [24]. All Co/Al samples, in addition to the peaks of the γ-Al_2_O_3_ support, present peaks at 2θ: 31°, 36.9° and 59.5° due to the formation of Co_3_O_4_, and these peaks exhibit increased intensity as the Co loading increases [17,24]. The formation of CoAl_2_O_4_ is also possible, as evidenced by the peaks at 2θ: 59.5° and 65.5°, which overlap the peaks of Co_3_O_4_ [17]. No important differences were observed between the diffractograms of the two synthesis methods. Unfortunately, the overlapping of the γ-Al_2_O_3_ and Co_3_O_4_ peaks at similar 2θ did not allow for size calculations of the formed Co_3_O_4_ particles, which is reported as an important parameter in the CO oxidation reaction [25]. The further incorporation of Pd (Figure 1b) into the Co/Al samples interestingly revealed one additional peak at 2θ: 33.8°, related to the formation of PdO, despite the low metal loading (0.5 wt.%) [17,18]. 

**Figure 2:**
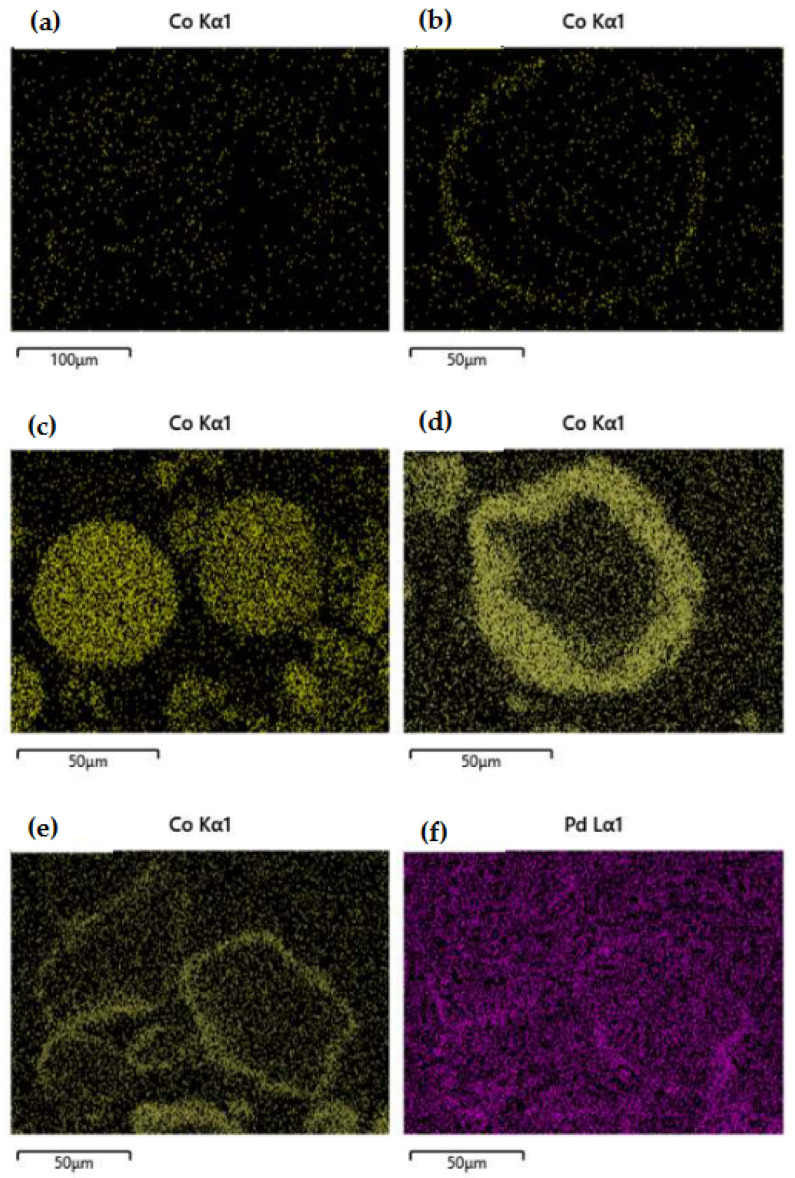
SEM images of (**a**) 1Co/Al-WI; (**b**) 1Co/Al-SI; (**c**) 5Co/Al-WI; (**d**) 5Co/Al-SI; (**e**,**f**) Pd(SI)-5Co/Al-SI.

The SEM images of selected samples are presented in Figure 2a–f. The differences between the two synthesis methods were investigated focusing on the preferential deposition of Co (yellow spots) and Pd (purple spots) over the alumina carrier. The image of the 1Co/Al sample, prepared with the wet impregnation method (Figure 2a), shows that Co is dispersed all over the alumina particle, while on the other side, the spray impregnation method (Figure 2b) achieves the deposition of Co on the outer surface of the alumina particle, creating the desired core–shell nanostructure. In the case of a higher Co content (5 wt.%), the deposition of the metal oxide on the outer surface of the alumina particle is even more obvious when applying the SI method than when applying the WI method (Figure 2c,d), where the cobalt is once again dispersed all over the alumina particle. Similarly, the incorporation of Pd via spray impregnation resulted in Pd deposition on the outer surface of Co on the alumina carrier, since the metal impregnation was performed successively (Figure 2e,f).

#### 3.1.3. Catalyst Reducibility (TPR-H_2_)

**Figure 3:**
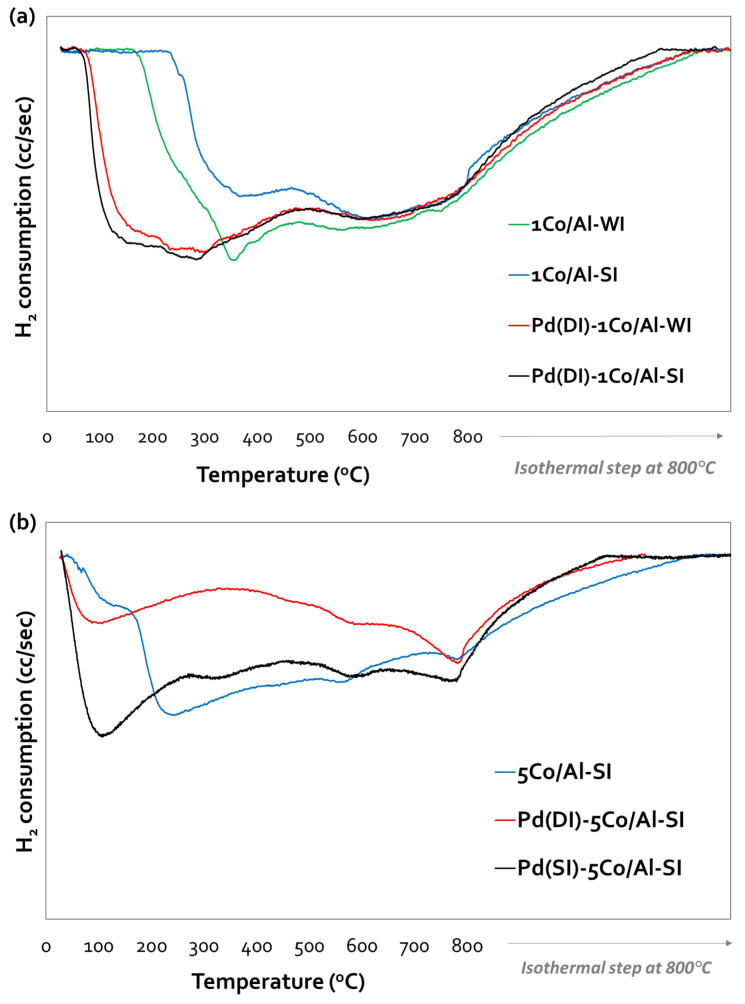

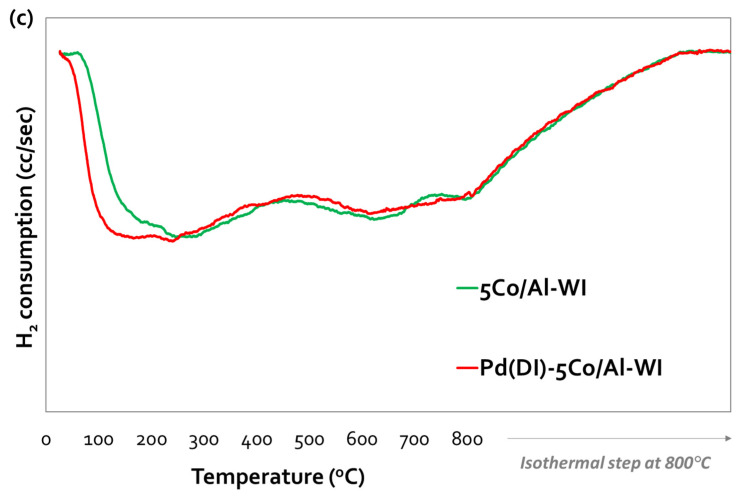
Reduction profiles of (**a**) Pd(DI)-1Co/Al-WI and -SI; (**b**) Pd(DI or SI)-5Co/Al-SI and (**c**) Pd(DI)-5Co/Al-WI samples.

The reducibility of all samples was investigated with TPR-H_2_, and the corresponding reduction profiles, for both synthesis methods, are presented in Figure 3a–c for (Pd)-1Co/Al, (Pd)-5Co/Al-SI and (Pd)-5Co/Al-WI, respectively. All the profiles present two main reduction areas: a low-temperature area (170–400 °C) and a high-temperature area (>400 °C). The first one is related to the reduction of Co_3_O_4_ to CoO, while the second one is due to the reduction of CoO to Co^0^ [26]. According to recent studies [27,28], it is possible that the high-temperature area is related to the reduction of CoAl_2_O_4_, which is more difficult to reduce at high temperatures (~800 °C) due to the strong interaction of Co with the alumina carrier [29]. The possible reactions (Equations (1)–(3)) that take place during the reduction of the Co/γ-Al_2_O_3_ samples are as follows [29]:

(1) $$Co_{3}O_{4}+H_{2}\;\to 3CoO+H_{2}O$$

(2) $$CoO+H_{2}\;\to Co+H_{2}O$$

(3) $$Co_{x}O_{y}-Al_{2}O_{3}+yH_{2}\;\to xCo+yH_{2}O+Al_{2}O_{3\;}$$

The Co/alumina samples (without Pd content) with 1 wt.% Co (Figure 3a) presented similar reduction profiles (despite using different synthesis methods), with two reduction areas: a peak at 170–400 °C and a broad peak at temperatures higher than 400 °C. These peaks were attributed to the step reduction of Co_3_O_4_ → CoO → Co^0^ [26]. The increase in Co content to 5 wt.% differentiated the TPR profiles of 5Co/Al-WI and 5Co/Al-SI (Figure 3b,c). For the WI method, the increase in Co content and the dispersion of the metal on the alumina surface presented two main reduction areas with broad peaks, starting from 60 °C. The second broad peak (at T > 400 °C) exhibited two maximums at 600 °C and 800 °C. Thus, the increase in cobalt content resulted in a stronger interaction between Co and the alumina carrier (possibly enhancing CoAl_2_O_4_ formation, which normally requires a much higher temperature than the one used in our synthesis procedure), which is more difficult to reduce [29]. On the other side, the SI method resulted in different reductive properties due to the deposition of Co on the outer surface of the alumina carrier, as revealed by the SEM analysis (Figure 2d). The main peak was centered at 240 °C, with a shoulder at 120 °C, while at higher temperatures (at T > 400 °C), the same two peaks appeared (as in the 5Co/Al-WI sample) but with a lower H_2_ consumption. This may imply that less Co-Al species formed when applying the SI method.

Impressively, the addition of Pd, even at a low loading (0.5 wt.%), affected the reducibility of the catalysts by shifting the reduction profiles to lower temperatures—initiating the reduction of Pd and/or Co oxides at room temperature—thus indicating a significant increase in the reducibility of the catalysts (Figure 3a,b). The Pd incorporation method used on both Co/Al-WI samples (with Co loadings of 1 and 5 wt.%) was the incipient wetness impregnation method (dry impregnation, DI), and their TPR profiles were similar but shifted to lower temperatures as a result of Pd addition (Figure 3a,c). No extra peak due to PdO was observed, probably due to the well-dispersed PdO particles and the interaction with Co/alumina, which facilitated the reduction of Co_3_O_4_ [30,31]. The same reduction profile was observed for Pd(DI)-1Co/Al-SI (Figure 3a). The incorporation of Pd into 5Co/Al-SI (Figure 3b) was studied by applying either incipient wetness impregnation or spray impregnation. Both catalysts presented a peak, with a maximum at 100 °C for Pd(DI)-5Co/Al-SI and at 108 °C for 0.5 Pd(SI)-5Co/Al-SI, shifting to lower temperatures (as compared to 240 °C for bare 5Co/Al-SI, with a shoulder at 120 °C). In this case, the impregnation method of Pd seemed to affect the reductive properties of the catalyst and especially H_2_ consumption; more H_2_ was consumed for the reduction of Pd(SI)-5Co/Al-SI than Pd(DI)-5Co/Al-SI. It is well known that noble metals like Pd, due to the H_2_ spillover effect, facilitate the reduction of the Co_3_O_4_ and Co-Al oxides formed from the interaction of cobalt with an alumina carrier [30].

### 3.2. CO Oxidation

**Figure 4:**
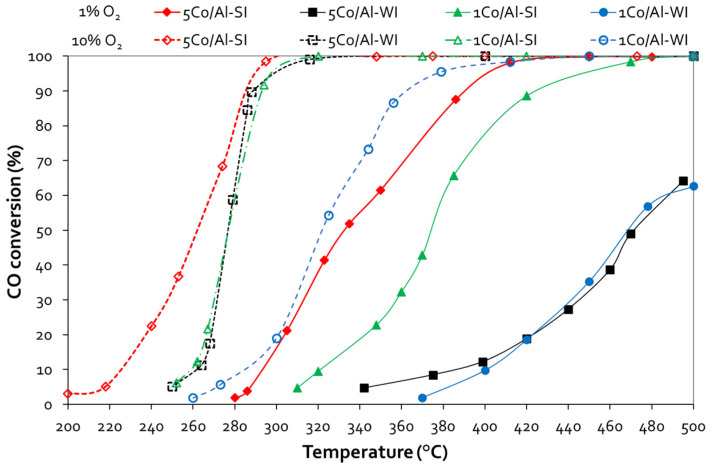
CO conversion vs. temperature plots for Co/γ-Al_2_O_3_ samples prepared with different methods and various Co loadings *(experimental conditions: 1 vol.% CO—1 or 10 vol.% O_2_, 40,000 h^−1^ GHSV)*.

Figure 4 shows the evaluation of all Co/γ-Al_2_O_3_ catalysts for the CO oxidation reaction. Under 1 vol.% O_2_ in the feed, the Co content (1 or 5 wt.%) did not affect the CO oxidation activity of the catalysts prepared with the conventional WI method. Thus, both the 1 and 5 wt.% Co/γ-Al_2_O_3_ samples presented ~64% CO conversion at 500 °C. However, for the SI catalysts, increasing the Co content enhanced the catalytic activity, while the 5Co/Al-SI sample achieved complete CO conversion at temperatures as low as 450 °C. A higher O_2_ concentration in the feed (10 vol.%) improved the performance of all catalysts. 5Co/Al-SI exhibited the highest CO oxidation activity, achieving 100% CO conversion from 300 °C, followed by 1Co/Al-SI, 5Co/Al-WI and 1Co/Al-WI. The SI synthesis method, that is, Co deposition on the outer surface of the alumina particle (as revealed by the SEM analysis), even at lower Co loading (1 wt.%), exhibited very similar catalytic performance to the 5Co/Al-WI sample, which, however, contained 5 times more Co than 1Co/Al-SI (during evaluation with 10 vol.% O_2_ in the feed). The light-off temperature (T_50_) and complete conversion temperature (T_90_) of all materials are listed in Table 2.

**Figure 5:**
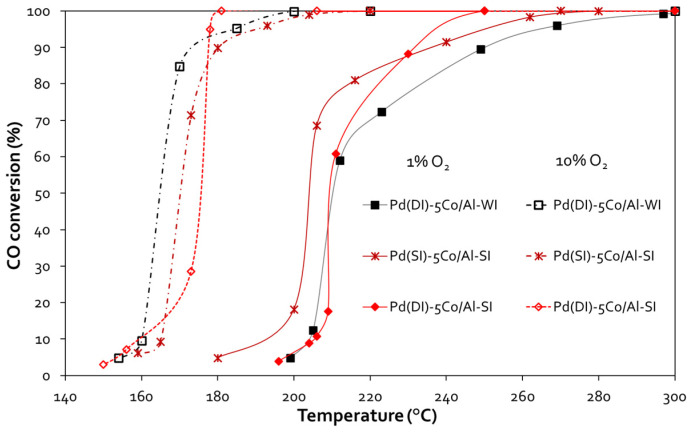
CO conversion vs. temperature plots for Pd-Co/γ-Al_2_O_3_ samples prepared with different methods *(experimental conditions: 1 vol.% CO—1 or 10 vol.% O_2_, 40,000 h^−1^ GHSV)*.

Next, the effect of Pd addition on the CO oxidation reaction was investigated, and the CO oxidation performance of the 0.5 wt.% Pd—5 wt.% Co/γ-Al_2_O_3_ samples is presented in Figure 5. In all cases, Pd addition improved the catalytic activity and shifted the activity curves to lower temperatures. More significantly, the temperature window of 100% CO oxidation was broadened from 500 °C up to 300 °C with 1 vol.% O_2_ in the feed and up to 200 °C with 10 vol.% O_2_ in the feed. In the first case (1 vol.% O_2_), Pd(SI)-5Co/Al-SI was the best catalyst, presenting T_50%_~204 °C and T_90%_ ~237 °C (for this sample, both metals, Pd and Co, were incorporated into γ-Al_2_O_3_ with the spray impregnation method), while the other two samples, Pd(DI)-5Co/Al-SI and Pd(DI)-5Co/Al-WI, presented T_50%_~211 °C and T_90%_~250 °C and 233 °C, respectively. The increase in O_2_ content in the feed (10 vol.%) shifted the light-off curves to even lower temperatures (ΔT~36–46 °C). In fact, Τ_50%_ ranged between 165 and 175 °C, while T_90%_~180°C for all the Pd-promoted samples. In all samples, Pd addition at 0.5 wt.% loading seemed to mask Co activity.

### 3.3. Methanol Oxidation

#### 3.3.1. Catalytic Evaluation

**Figure 6:**
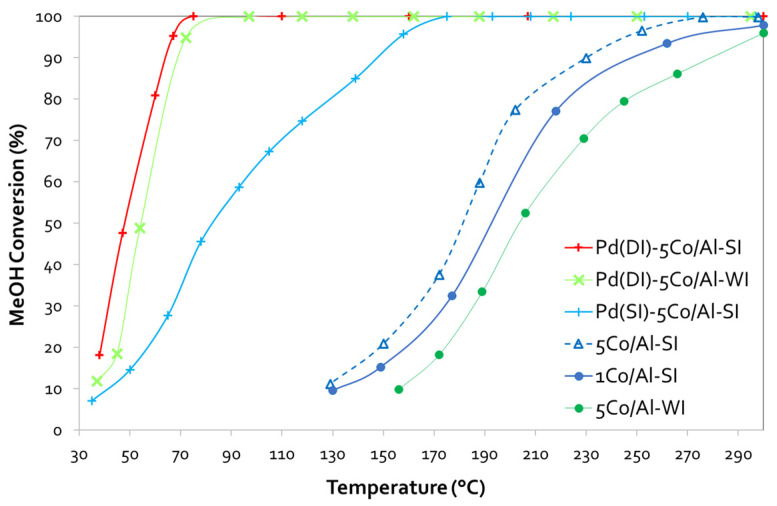
MeOH conversion vs. temperature plots for (Pd)-Co/γ-Al_2_O_3_ samples prepared with different methods and various Co loadings *(experimental conditions: 0.1 vol.% CH_3_OH—0.1 vol.% O_2,_ 40,000 h^−1^ GHSV)*.

Figure 6 presents the curves of methanol conversion with decreasing temperature on all Co/alumina and Pd-Co/alumina catalysts, while Table 3 summarizes the catalytic efficiency (T_50_ and T_90_ values) of the tested samples. 

Starting with the conventionally prepared WI of the Co/γ-Al_2_O_3_ sample, we only observed a limited efficiency, despite the fact that we selected a high (5 wt.%) Co-based material. On the contrary, when testing the samples prepared with the SI approach, we noticed a slightly enhanced performance, even in the case of only 1 wt.% Co, as both 1 and 5 wt.% loaded catalysts exhibited an activity curve that shifted to lower temperatures (~10 °C and 23 °C lower T_50%_ for the 1 and 5 wt.% Co/γ-Al_2_O_3_-SI samples, respectively, as compared with the conventional 5Co/Al-WI sample). Pd addition significantly boosted the catalytic performance, achieving 82 °C, 166 °C or even 209 °C lower T_90%_ than the same samples (5Co/Al-SI or 5Co/Al-WI, respectively) without Pd. In this case, the pathway of Pd addition seems very important, leading to totally different behavior. In detail, T_50_ shifted from 204 °C for 5Co/Al-WI to 54 °C for the Pd(DI)-5Co/Al-WI sample, from 181 °C to 83 °C for the Pd(SI)-5Co/Al-SI sample and to 48 °C for the Pd(DI)-5Co/Al-SI sample. It is worth highlighting the Pd-based 5Co/Al catalyst prepared using DI for the incorporation of Pd into both 5Co/Al-WI and -SI had better performance than Pd(SI)-5Co/Al-SI, where Pd was impregnated with the SI method.

Undoubtedly, during the evaluation of the catalysts for MeOH oxidation, the conversion plots of the Co/alumina catalysts prepared with different methods and different Co loadings revealed that both parameters (the synthesis method and Co loading) affected the activity of the Co-based catalysts. As evidenced by SEM, the spray impregnation method resulted in the deposition of Co_3_O_4_ on the outer surface of the alumina particles, and in this way, more active phase was available for the oxidation of MeOH. On the other side, the wet impregnation method resulted in the dispersion of Co all over the alumina particles, and it is likely that not all of the active phase of cobalt oxide was available for the oxidation reaction, leading to the 5Co/Al-WI sample having a lower catalytic activity than 1Co/Al-SI, in spite of the fact that the former had a five times higher cobalt content. In addition, the Pd incorporation method significantly affected the methanol oxidation performance of the Pd-5Co/Al-SI samples. Based on the SEM analysis, the successive impregnation of Pd with the spray impregnation method, after the initial impregnation of Co also with SI, resulted in the deposition of Pd on the formed Co oxides creating a second layer of PdO (Figure 2f). In this way, some of the active phase of Co_3_O_4_ was probably covered with PdO and was not available for the oxidation of MeOH, while the dispersion of PdO was limited. On the contrary, the incorporation of Pd with the DI method resulted in the better dispersion of PdO all over the Co/Al catalyst surface, providing available Co_3_O_4_ active sites and probably a stronger interaction between Pd and Co/Al. 

**Figure 7:**
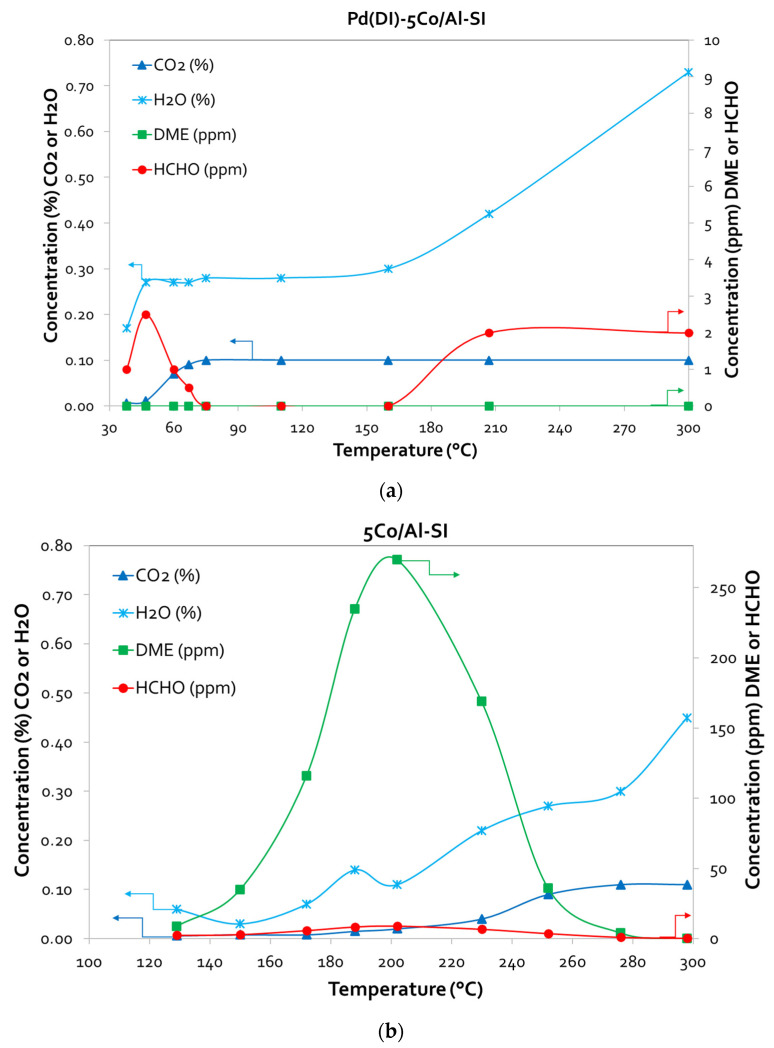
Product concentration vs. temperature plots for (**a**) Pd(DI)-5Co/ Al-SI and (**b**) 5Co Al-SI samples during MeOH conversion (experimental conditions: 0.1 vol.% CH_3_OH—0.1 vol.% O_2_, 40,000 h^−1^ GHSV).

The main products of the methanol oxidation reaction on the Co/Al catalysts were CO_2_ and H_2_O in the high-temperature area (250–300 °C), while at lower temperatures (125–250 °C), both products decreased, and DME increased. When Pd was also incorporated into the catalyst surface, besides CO_2_ and H_2_O, the main products monitored, traces of HCHO were also detected at low temperatures, without the formation of DME. All of the above are selectively presented for two representative samples, Pd(DI)-5Co/Al-SI and 5Co/Al-SI, in Figure 7a and Figure 7b, respectively. 

#### 3.3.2. Methanol Desorption Studies (TPD-MeOH)

**Figure 8:**
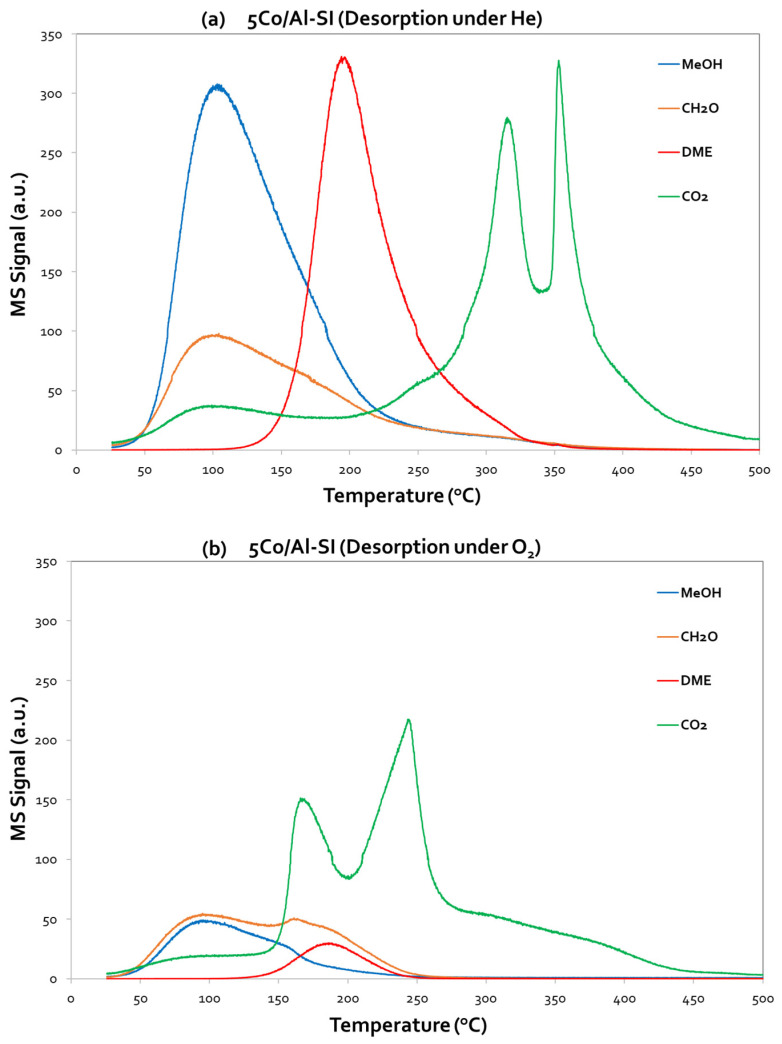
Methanol desorption (TPD-MeOH) profiles for 5Co/Al-SI under (**a**) He flow and (**b**) O_2_ flow.

**Figure 9:**
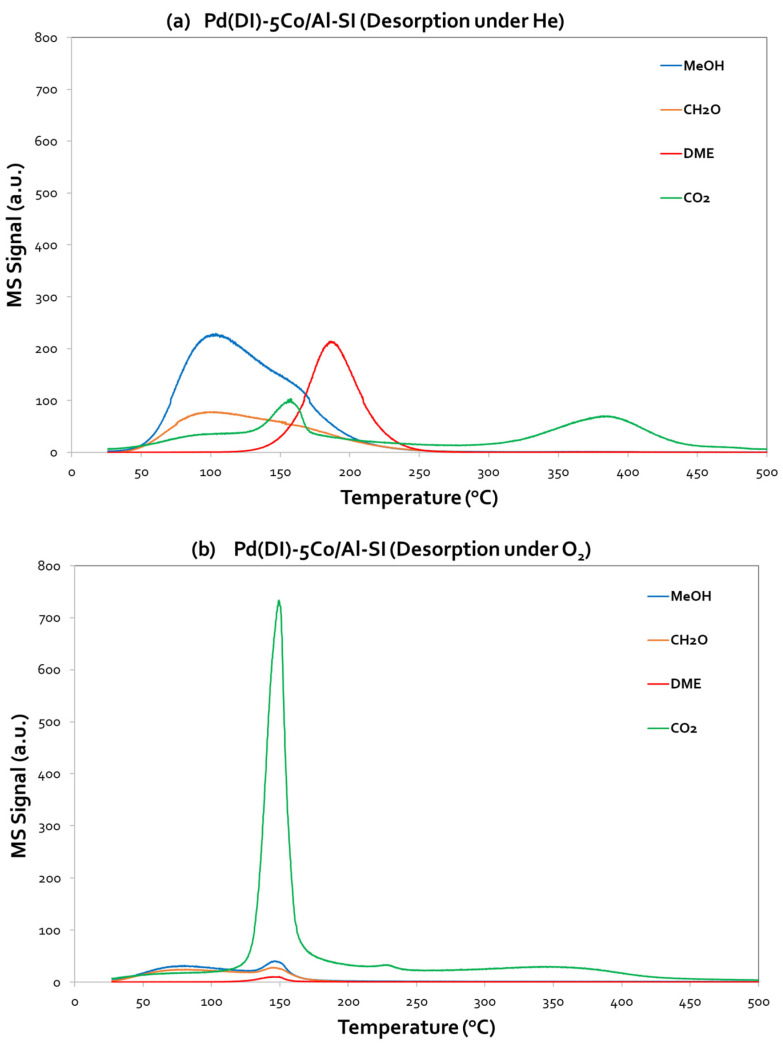
Methanol desorption (TPD-MeOH) profiles for Pd(DI)-5Co/Al-SI under (**a**) He flow and (**b**) O_2_ flow.3.3.3. Catalyst basicity (TPD-CO_2_).

The TPD-MeOH profiles of 5Co/Al-SI and Pd(DI)-5Co/Al-SI are presented in Figure 8a,b and Figure 9a,b, additionally varying the desorption atmosphere, which was under He and/or oxygen flow, respectively. Both figures evidence the desorption (corresponding MS signals) of MeOH, CO_2_, HCHO and DME.

Starting from 5Co/Al-SI (Figure 8), it is obvious that MeOH desorption initiated almost at room temperature and presented a maximum at ~95–100 °C under both desorption steps (He and O_2_). However, the intensity of the MeOH peaks was different; a higher intensity was observed under He desorption, which suggests that methanol was weakly adsorbed on the catalyst surface and thus desorbed in the gas phase. However, under an O_2_ atmosphere, the adsorbed methanol reacted with the oxygen, and, thus, less desorbed MeOH was measured. Besides the MeOH signal, CO_2_, HCHO and DME were also monitored (desorbed), as presented in Figure 8a,b.

Under He desorption, the 5Co/Al-SI catalyst presented a low ability to oxidize methanol using oxygen from the Co-Al surface, instead resulting in the partial oxidation of methanol to HCHO and CO_2_ (maximum at T~100 °C). At higher temperatures (T~200 °C), methanol was dehydrated to DME, while at even higher temperatures (T > 200 °C), a double CO_2_ peak was observed, centered at 315 °C and 350 °C, due to the oxidation of both MeOH and DME from the surface oxygen. Under O_2_ desorption, the signals of HCHO and DME decreased, while CO_2_ maintained the double peak; however, it shifted to lower temperatures (maximum at 170 °C and 240 °C). In this case, methanol oxidation to CO_2_ took place. The CO_2_ desorption peaks were assigned to active sites based on the desorption temperature. The active sites that produce the highest peak of CO_2_, in the low-temperature area, are responsible for the higher methanol oxidation activity [3]. 

However, concerning the Pd-based catalyst, Pd(DI)-5Co/Al-SI (Figure 9a,b), it is worth highlighting the high intensity of the CO_2_ peak, centered at 150 °C, under O_2_ desorption. Thus, CO_2_ was the main signal, and the adsorbed MeOH was completely oxidized to CO_2_. Even under the He desorption step, the same CO_2_ peak centered at 150 °C (with a lower intensity than the signal under O_2_ desorption) appeared in the TPD profiles of the Pd(DI)-5Co/Al-SI catalyst, implying that the addition of Pd facilitated the deep oxidation of methanol to CO_2_. The results from the TPD-MeOH study are in agreement with the methanol oxidation activity evaluation results of the investigated catalytic materials. During the MeOH oxidation reaction on the Pd-based catalysts, the products of the reaction were CO_2_ and H_2_O, while for the Co/Al catalysts, DME was mainly detected in the temperature area~125–275 °C.

**Figure 10:**
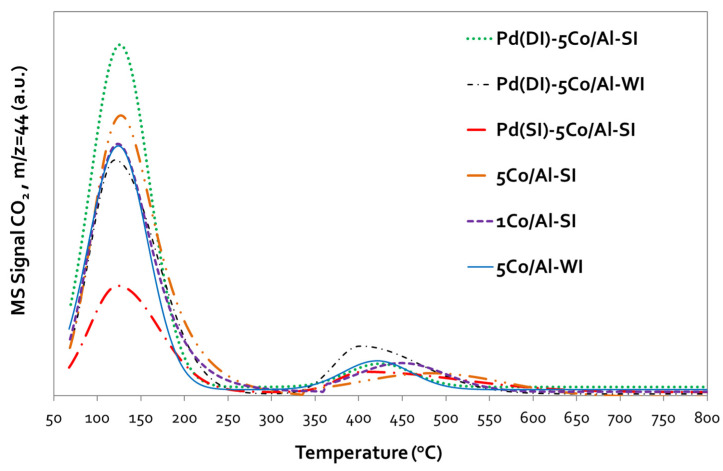
TPD-CO_2_ profiles of 5Co/Al and Pd-based 5Co/Al samples prepared with different methods.

The basicity of all samples was investigated with TPD-CO_2_, and the corresponding desorption profiles are provided in Figure 10, investigating the effect of both the synthesis technique and Pd presence/addition. All profiles present two main desorption peaks: a low-temperature area (70–300 °C) attributed to weak/medium basic sites and a high-temperature area (300–600 °C) related to strong basic sites. As obvious in Table 4, the basic properties varied according to both the synthesis method and the (pathway of) Pd promotion.

The approach of Co incorporation with SI increased the basicity, actually enhancing the weak basic sites. Similar basicity was shown by 5Co/Al-WI and 1Co/Al-SI, despite the different Co contents; even catalytically small differences were observed, indicating the superiority of the SI method. However, Pd promotion led to a higher basicity only in the case of Pd(DI)-5Co/Al-SI, significantly increasing the weak basic sites (25% more than the weak basic sites of the initial 5Co/Al-SI sample). Impressively, this was not the case when Pd was impregnated on the same Co-based material but following the SI technique (Pd(SI)-5Co/Al-SI sample). Conversely, in that case, Pd incorporation caused a significant decrease in the sample’s basicity, reducing the weak basic sites by ~59%, as compared with the same Co-based material before Pd addition. It is suggested [32] that a higher basicity can enhance the catalytic efficiency of the materials studied by changing the adsorption–desorption equilibria of the adsorbed species on the catalytic surface. Badlani and Wachs et al. [33] revealed that the methanol oxidation product distribution at low conversions reflects the nature of the surface active sites on metal oxides since redox sites primarily yield HCHO, acidic sites yield DME, and basic sites yield CO_2_.

## 4. Discussion

The investigated Co/γ-Al_2_O_3_ materials are very promising catalysts for CO and MeOH oxidation reactions. Both reactions require high oxygen mobility of Co_3_O_4_ on the alumina carrier, which was tested in TPR-H_2_ experiments (Figure 3). The reduction profiles revealed the reduction of Co_3_O_4_ to CoO at 170–400 °C, while at temperatures higher than 400 °C, CoO was further reduced to Co^0^. An increase in Co loading (from 1 to 5 wt.%) shifted the profiles to lower temperatures (more easily reducible Co_3_O_4_ species) and formed Co species, which exhibited stronger interactions with the alumina carrier (CoAl_2_O_4_) and are not desirable for oxidation reactions. However, the SI method formed less of these kinds of Co-Al species, and the addition of Pd further improved the catalyst reducibility (initiating the reduction of the Pd–Co species, even at room temperature).

The method of Co impregnation via SI resulted in the deposition of Co oxides on the outer surface of alumina particles, and the active phase of Co was available for the oxidation reactions. Even 5 times less Co content (1Co/Al-SI vs. 5Co/Al-WI) presented the same CO oxidation performance, while for methanol oxidation, the SI method showed higher catalytic efficiency. Concerning the CO oxidation reaction (Figure 4 and Figure 5), all Pd-based 5Co/Al catalysts presented more or less similar activity, with T_90_ at 180 °C and T_50_ ranging between 165 and 175 °C. For MeOH oxidation (Figure 6 and Figure 7), despite the good reducibility that the Pd-5Co/Al catalysts displayed, the Pd incorporation method seemed to be an important synthesis parameter for the studied reaction. Based on the SEM analysis (Figure 2), the incorporation of Pd into 5Co/Al-SI with the SI approach resulted in the deposition of less dispersed Pd on the outer surface of Co on the alumina carrier; thus, not all Co_3_O_4_ active phases were available for the oxidation of MeOH. On the contrary, the dry impregnation of Pd into the 5Co/Al-SI catalyst resulted in a higher dispersion of Pd on the catalyst surface, without covering the Co_3_O_4_ active phase or limiting the Pd-Co interaction.

MeOH oxidation is a more complicated reaction than CO oxidation, which involves several reaction steps; thus, besides the cobalt oxidation state (Co_3_O_4_, CoO), the reaction mixture (absence or presence of O_2_) is also crucial for the oxidation mechanism. Based on the literature [33,34], the mechanism of methanol adsorption begins with the formation of methoxy species CH_3_O (Equation (4)), which are transformed to HCHO (Equation (5)): partial oxidation-absence of O_2_) or CO_2_ (Equation (6)): complete oxidation-presence of O_2_). In addition, DME is probably formed from weakly adsorbed methoxy species, according to the reaction (Equation (7)) proposed by Akarmazyan et al. [35].

(4) $$CH_{3}OH_{\left(g\right)}+OH_{\left(ads\right)}\;↔CH_{3}O_{\left(ads\right)}+H_{2}O_{\left(ads\right)}$$

(5) $$CH_{3}OH\;\to HCHO+H_{2}$$

(6) $$CH_{3}OH+\frac{3}{2}\;O_{2}\;\to CO_{2}+2H_{2}O$$

(7) $$2CH_{3}O_{\left(ads\right)}\;↔CH_{3}OCH_{3}+OH_{\left(surf\right)}$$

According to the catalytic evaluation results and desorption experiments (Figure 8 and Figure 9) of the present study, methanol oxidation seems to follow the proposed mechanism. The main products of the Co/Al catalysts in CH_3_OH oxidation were CO_2_ and H_2_O (at T: 250–300 °C), while at lower temperatures (<250 °C), the main by-product was DME. The Pd-based catalysts oxidized methanol to CO_2_ and H_2_O, without the formation of DME. Traces of HCHO were also detected at low temperatures. Temperature-programmed desorption studies revealed the different oxidation mechanisms of the 5Co/Al-SI and Pd(DI)-5Co/Al-SI catalysts. Based on the desorbed signals, it seems that the 5Co/Al-SI catalyst promoted the dehydration reaction to DME (Equation (7)), while the Pd-based catalyst promoted the oxidation reaction to CO_2_ and H_2_O (Equation (6)). Moreover, for deep methanol oxidation, Luo et al. [3] studied the basicity of Pd/Al-Ce_x_Zr_1-x_O_2_ catalysts and proved that more strong basic sites enhance the catalysts’ activity. The TPD-CO_2_ results (Figure 10) and the integration of the peaks (Table 4) are in agreement with the superior performance of the Co/Al catalytic samples with Pd applied via the dry impregnation method (prepared via either the WI or SI method).

## 5. Conclusions

The activity of Co/γ-Al_2_O_3_ and 0.5 wt.% Pd-promoted Co/γ-Al_2_O_3_ catalysts for CO and MeOH oxidation was investigated, and different Co loadings (1 and 5 wt.%) and synthesis methods (wet impregnation and spray impregnation) were applied for the preparation of the catalysts under study. The results revealed that both factors affected the catalysts’ performance; an increase in Co loading improved the catalysts’ oxidation activity for both reactions, especially for the catalysts prepared with the SI method. The superiority of these samples was based on the formation of core–shell nanostructures, where most Co_3_O_4_ was deposited on the outer surface of the γ-Al_2_O_3_ carrier. For both reactions, the 1 wt.% Co loading prepared with the SI method showed higher activity than the 5 wt.% Co loading prepared with the WI method. Further promotion with a low Pd content applied via either incipient wetness impregnation or spray impregnation increased the catalysts’ reducibility and improved the catalysts’ performance for both reactions. For CO oxidation, all Pd-based catalysts presented similar performance, while for MeOH oxidation, the Pd incorporation method determined the catalytic performance. The impregnation of Pd with the DI method resulted in a better dispersion of PdO all over the Co/γ-Al_2_O_3_ catalyst surface, maintaining available Co active sites and the Pd-Co interaction. In addition, investigating the mechanism of the MeOH oxidation reaction in MeOH desorption experiments revealed that the Co/Al catalysts promoted the partial oxidation of MeOH to formaldehyde (HCHO) and dehydration to dimethyl ether (DME), while the Pd-based Co/Al catalysts enhanced the desired complete oxidation to CO_2_ and H_2_O. These results are in agreement with the catalytic evaluation tests, where the Pd(DI)-5Co/Al-SI catalyst exhibited the optimum performance, achieving a light-off temperature of 48 °C (T_50_) and a complete conversion temperature of 64 °C (T_90_).

## Figures and Tables

**Table 1:** Textural properties.

Sample	Co, wt.%	Pd, wt.%	Surface Area, m^2^/g	Pore Volume, cm^3^/g	Pore Size, nm
γ-Al_2_O_3_	-	-	226.0	0.65	11.5
1Co/Al-WI	1.06	-	182.2	0.64	13.0
1Co/Al-SI	1.05	-	170.0	0.64	13.2
5Co/Al-WI	5.46	-	148.6	0.59	13.0
5Co/Al-SI	5.14	-	158.5	0.57	11.6
Pd(DI)-1Co/Al-WI	1.06	0.58	159.7	0.63	13.5
Pd(DI)-1Co/Al-SI	1.05	0.60	152.9	0.63	13.6
Pd(DI)-5Co/Al-WI	5.46	0.58	140.1	0.60	13.4
Pd(DI)-5Co/Al-SI	5.46	0.60	157.7	0.54	10.9
Pd(SI)-5Co/Al-SI ^1^	5.14	0.50	144.0	0.56	11.9

^1^ Both metals, Co and Pd, were impregnated by successively applying the SI method.

**Table 2:** T_50_ and T_90_ values of CO oxidation.

Sample	CO Oxidation			
	1 vol.% O_2_		10 vol.% O_2_	
	T_50_, °C	T_90_, °C	T_50_, °C	T_90_, °C
1Co/Al-WI	469	-	322	365
1Co/Al-SI	375	427	278	293
5Co/Al-WI	472	-	277	-
5Co/Al-SI	333	392	262	289
Pd(DI)-5Co/Al-WI	211	250	165	177
Pd(DI)-5Co/Al-SI	211	233	175	178
Pd(SI)-5Co/Al-SI	204	237	170	180

**Table 3:** T_50_ and T_90_ of MeOH oxidation.

Sample	MeOH Oxidation	
	T_50_, °C	T_90_, °C
1Co/Al-SI	193	252
5Co/Al-WI	204	279
5Co/Al-SI	181	230
Pd(DI)-5Co/Al-WI	54	70
Pd(DI)-5Co/Al-SI	48	64
Pd(SI)-5Co/Al-SI	83	148

**Table 4:** Basicity of Co/Al and Pd-based 5Co/Al samples prepared with different methods and various Co loadings.

Sample	Basicity, μmol CO_2_/g Catalyst		
	Weak/Medium Sites	Strong Sites	Total
1Co/Al-SI	15.2	2.5	17.7
5Co/Al-WI	15.7	2.2	17.9
5Co/Al-SI	16.9	2.1	19.1
Pd(DI)-5Co/Al-WI	14.9	3.6	18.5
Pd(DI)-5Co/Al-SI	21.2	2.1	23.3
Pd(SI)-5Co/Al-SI	7.0	2.4	9.4

## Data Availability

Data are contained within the article.
